# End-to-End provenance representation for the understandability and reproducibility of scientific experiments using a semantic approach

**DOI:** 10.1186/s13326-021-00253-1

**Published:** 2022-01-06

**Authors:** Sheeba Samuel, Birgitta König-Ries

**Affiliations:** 1 Heinz-Nixdorf Chair for Distributed Information Systems, Friedrich Schiller University, Jena, Germany; 2 Michael Stifel Center Jena, Jena, Germany

**Keywords:** Provenance, Reproducibility, Semantic web, Ontology, Jupyter notebooks, Experiments

## Abstract

**Background:**

The advancement of science and technologies play an immense role in the way scientific experiments are being conducted. Understanding how experiments are performed and how results are derived has become significantly more complex with the recent explosive growth of heterogeneous research data and methods. Therefore, it is important that the provenance of results is tracked, described, and managed throughout the research lifecycle starting from the beginning of an experiment to its end to ensure reproducibility of results described in publications. However, there is a lack of interoperable representation of end-to-end provenance of scientific experiments that interlinks data, processing steps, and results from an experiment’s computational and non-computational processes.

**Results:**

We present the “REPRODUCE-ME” data model and ontology to describe the end-to-end provenance of scientific experiments by extending existing standards in the semantic web. The ontology brings together different aspects of the provenance of scientific studies by interlinking non-computational data and steps with computational data and steps to achieve understandability and reproducibility. We explain the important classes and properties of the ontology and how they are mapped to existing ontologies like PROV-O and P-Plan. The ontology is evaluated by answering competency questions over the knowledge base of scientific experiments consisting of computational and non-computational data and steps.

**Conclusion:**

We have designed and developed an interoperable way to represent the complete path of a scientific experiment consisting of computational and non-computational steps. We have applied and evaluated our approach to a set of scientific experiments in different subject domains like computational science, biological imaging, and microscopy.

**Supplementary Information:**

The online version contains supplementary material available at (10.1186/s13326-021-00253-1).

## Introduction

Scientific experiments play a key role in new inventions and in extending the world’s knowledge. The way science is being done has greatly changed with the emergence of technologies and instruments which can produce and process big data. Several existing and new challenges have come into the picture with the increasing magnitude of data being produced in experiments and the increasing complexity to track how experimental results are derived. The “Reproducibility Crisis” is one such challenge faced in this modern era driven by computational science [1–6].

According to NIST [7], a scientific experiment is said to be *reproducible* if the experiment can be performed to get the same or similar (close-by) results by a different team using a different experimental setup. The reproducibility crisis was brought to the scientific community’s attention by the survey conducted by Nature in 2016 among 1576 scientists. This survey showed that 70% of the researchers have tried and failed to reproduce other researcher’s experiments [4]. The reproducibility crisis is currently faced by various disciplines from life sciences [1] to artificial intelligence [5]. Different measures and research works are being conducted to tackle this problem to enable reproducibility. Provenance-based tools and vocabularies have been introduced to address the issue. Journals, for example, Nature [8], are making it compulsory to ensure that the data and associated materials used for experiments mentioned in the publications are findable and accessible. The FAIR principles introduced in this regard in 2016 define the metrics for findability, accessibility, interoperability, and reuse of data [9]. It is important that these measures are taken not only when the scientific papers are published, but also throughout the research lifecycle from the acquisition of data to the publication of results [10]. To ensure end-to-end reproducibility, it is important to enable end-to-end provenance management of scientific experiments. At the same time, the *provenance*, the source or origin of an object, needs to be represented in an interoperable way for the understandability and reuse of data and results.

In this article, we aim to provide reproducibility measures from the beginning of an experiment to the publication of its results. To do so, we combine the concepts of provenance [11] and semantic web technologies [12] to represent the complete path of a scientific experiment. There are many challenges to track the provenance of results to represent this complete path. They include the lack of a link between steps, data and results from different data sources, a lack of common format to share end-to-end provenance of results, and loss of data and results from different trials conducted for an experiment. To address these challenges, we present a standard data model, “REPRODUCE-ME”, to represent the complete path of a scientific experiment including its non-computational and computational parts.

In the following section, we discuss the related work in this area. In the Results section, we present each of our contributions. The research methodology is presented in the Methods section. This is followed by the evaluation and discussion of our results. Finally, we conclude the work by providing insights into our future work.

## Background & related work

**Fig. 1:**
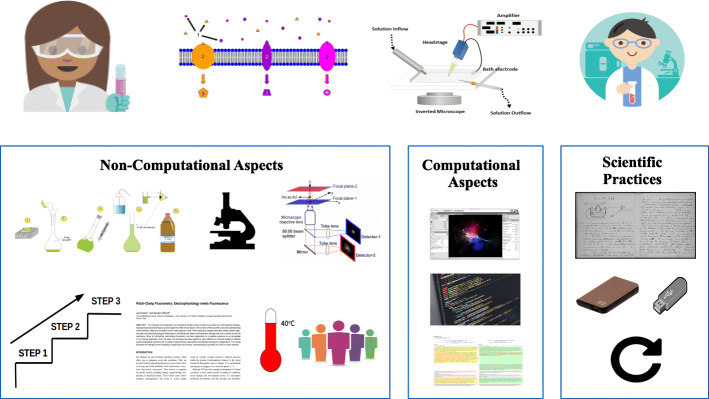
An overall view of the scientific experiments and practices

The prerequisite to designing and developing an end-to-end provenance representation of scientific experiments arises from the requirements collected from interviews we conducted with scientists working in the Collaborative Research Center (CRC) ReceptorLight [13], as well as from a workshop conducted to foster reproducible science [14]. The participating scientists come from different disciplines including Biology, Computer Science, Ecology, and Chemistry. We also conducted a survey addressed to researchers from different disciplines to understand scientific experiments and research practices for reproducibility [6]. The detailed insights from these meetings and the survey helped us to understand the different scientific practices followed in their experiments and the importance of reproducibility when working in a collaborative environment as described in [13]. Figure 1 provides an overall view of the scientific experiments and practices. *Reproducibility* and related terms used throughout this paper are clearly defined in the Results section. A *scientific experiment* consists of non-computational and computational data and steps. Computational data is generated from computational tools like computers, software, scripts, etc. Non-computational data and steps do not involve computational tools. Activities in the laboratory like preparation of solutions, setting up the experimental execution environment, manual interviews, and observations are examples for non-computational activities. Measures taken to reproduce a *non-computational step* are different from those for a *computational step*. One of the key requirements to reproduce a computational step is to provide the script/software along with the data. However, for non-deterministic computational tasks, providing software and data alone is not sufficient. The *reproducibility* of a non-computational step, on the other hand, depends on various factors like the availability of experiment materials (e.g., animal cells or tissues) and instruments, the origin of the materials (e.g., distributor of the reagents), human and machine errors, etc. Hence, it is important to describe non-computational steps in sufficient detail for their reproducibility [1].

The conventional way of recording the experiments in hand-written lab notebooks is still in use in fields like biology and medicine. This creates a problem when researchers leave projects and join new projects. To understand the previous work conducted in a research project, all the information regarding the project including previously conducted experiments along with the trials, analysis, and results must be available to the new researchers in a reusable way. This information is also required when scientists are working in big collaborative projects. In their daily research work, a lot of data is generated and consumed through computational and non-computational steps of an experiment. Different entities like devices, procedures, protocols, settings, computational tools, and execution environment attributes are involved in experiments. Several people play various roles in different steps and processes of an experiment. The outputs of some non-computational steps are used as inputs to the computational steps. Hence, an experiment must not only be linked to its results but also to different entities, people, activities, steps, and resources. Therefore, it is important that the complete path towards results of an experiment is shared and described in an interoperable manner to avoid conflicts in experimental outputs.

### Related work

Various research works are being done in different disciplines to describe provenance to enable the reproducibility of results. We analyze the current state of the art approaches ensuring the computational and non-computational aspects of the reproducibility.

**Provenance Models**

Ram et al. introduce the W7 model to represent the semantics of data provenance [15]. It presents seven different components of provenance and how they are related to each other. The models defines provenance as an n-tuple: P = (WHAT, WHEN, WHERE, HOW, WHO, WHICH, WHY, OCCURS_AT, HAPPENS_IN, LEADS_TO, BRINGS_ABOUT, IS_USED_IN, IS_BECAUSE_OF) where P is the provenance; WHAT denotes the sequence of events that affect the data object; WHEN, the set of times of the event; WHERE, the set of locations of the event; HOW, the set of actions that lead to the events; WHO, the set of agents involved in the events; WHICH, the set of devices and WHY, the set of reasons for the event. OCCURS_AT is a collection of pairs (e, t) where e belongs to WHAT and t belongs to WHEN. HAPPENS_IN represents a collection of pairs (e, l) where l represents a location. LEADS_TO is a collection of pairs (e, h) where h denotes an action that leads to an event e. BRINGS_ABOUT is a collection of pairs (e, *a*_1_, *a*_2_,..*a*_*n*_) where *a*_1_, *a*_2_,..*a*_*n*_ are agents who cooperate to bring about an event e. IS_USED_I is a collection of pairs (e, *d*_1_, *d*_2_,..*d*_*n*_) where *d*_1_, *d*_2_,..*d*_*n*_ denotes devices. IS_BECAUSE_OF is a collection of pairs (e, *y*_1_, *y*_2_,..*y*_*n*_) where *y*_1_, *y*_2_,..*y*_*n*_ denotes the reasons [15]. This model provides the concepts to define the provenance in the context of events and actions.

Another provenance model that inspired our work is the PRIMAD [16] model. It describes a list of variables that could be changed or remain the same when trying to reproduce a study. They are: **P**latform, **R**esearch Objective, **I**mplementation, **M**ethod, **A**ctor, and **D**ata. The authors provide how a change in each variable of the PRIMAD model results in various types of reproducibility and the gain delivered to a computational experiment. For example, if only the platform is changed and keeping the rest same, then the reproducibility study tests the portability of an experiment. When none of the variables in the PRIMAD data model are changed with the aim to verify whether the results are consistent, then the experiment is said to be repeated.

Another standard data model, PROV-DM, was introduced after the First, Second and Third Provenance Challenges by the W3C working group [17]. The PROV-DM is a generic data model to describe and interchange provenance between systems. It has a modular design with six components: Entities and Activities, Derivation of Entities, Agents and Responsibilities, Bundles, Properties that link entities, and Collections. We bring together the various variables defined in these models to describe the different aspects of provenance of scientific experiments.

**Ontologies**

Ontologies are formal, explicit specifications of shared conceptualizations [18]. They play a key role in the Semantic web to support interoperability and to make data machine-readable and machine-understandable. Various provenance models and ontologies have been introduced in different domains, ranging from digital humanities to biomedicine [19–22]. PROV-O, a W3C recommendation, is a widely used ontology to provide the interoperable interchange of provenance information among heterogeneous applications [23]. The PROV-O ontology is the encoding of PROV-DM in the OWL2 Web Ontology Language.

Many provenance models are developed mostly focusing on scientific workflows like DataONE [24], ProvOne [25], and OPMW [26]. A Scientific Workflow is a complex set of data processes and computations usually represented as a directed acyclic graph with nodes representing tasks and edges representing dependencies between tasks [27]. These data-oriented workflows are constructed with the help of a Scientific Workflow Management System (SWfMS) [28]. Different SWfMSs have been developed for different use cases and domains [28–32]. Most of the SWfMSs provide provenance support by capturing the history of workflow executions. These systems focus on the computational steps of an experiment and the experimental metadata are not linked to the results. Even though P-Plan [33] is developed to model the executions of scientific workflows, the general terms provided in it make it possible to use it in other contexts as well. These initiatives [24–26] reuse and extend PROV to capture retrospective and prospective provenance of scientific workflows like channel and port-based scientific workflows, complex scientific workflows with loops and optional branches, and specificities of particular SWfMSs [34]. Despite the provenance modules present in these systems, there are currently many challenges in the context of reproducibility of scientific workflows [34, 35]. Workflows created by different scientists are difficult for others to understand or re-run in a different environment, resulting in *workflow decay*s [35]. The lack of interoperability between scientific workflows and the steep learning curve required by scientists are some of the limitations according to the study of different SWfMSs [34]. The Common Workflow Language [36] is an initiative to overcome the lack of interoperability of workflows. Though there is a learning curve associated with adopting workflow languages, this ongoing work aims to make computational methods reproducible, portable, maintainable and shareable.

The Workflow-centric Research Objects consists of four ontologies to support aggregation of resources and domain-specific workflow requirements [37]. The complete path for a scientific workflow could be described using Research Objects because they represent the resources, the prospective and retrospective provenance and the evolution of workflows. We apply the idea in the context of scientific experiments inspired by this work. In our approach, we focus on a vocabulary which provides general provenance terms that can be used and applied to conceptualize the scientific experiments.

In addition to the general-purpose vocabularies to model provenance, many ontologies are developed to capture the requirements of individual domains [38–41]. The EXPO ontology [41] describes knowledge about experiment design, methodology, and results. It focuses more on the design aspects of an experiment and does not capture the execution environment and the execution provenance of an experiment. Vocabularies like voiD [42] and DCAT [43] describe linked datasets and data catalogs, respectively. The Ontology for Biomedical Investigations [44] is another ontology developed as a community effort to describe the experimental metadata in biomedical research and has been widely adopted in the biomedical domain. SMART Protocols (SP) [45] is another ontology-based approach to represent experimental protocols. Ontologies such as EXPO, OBI, SWAN/SIOC provide vocabularies that allow the description of experiments and the resources that are used within them. However, they do not use the standard PROV model thus preventing the interoperability of the collected data.

One of our use-cases includes the semantic representation of imaging experiments to describe how images are obtained and which instruments and settings are used for their acquisition. Kume et al. [46] present an ontology to describe imaging metadata for the optical and electron microscopy images. They construct a Resource Description Framework (RDF) schema from the Open Microscopy Environment (OME) [47] data model. This work is close to ours but the use of PROV to represent the imaging metadata in our work provides the additional benefit of interoperability.

However, these ontologies do not directly provide the features to fully represent the complete path of a scientific experiment. There exists a gap in solutions as they do not interlink the data, the steps and the results from both the computational and non-computational processes of a scientific experiment. Hence, it is important to extend the current approaches and at the same time, reuse their rich features to support the reproducibility and understandability of scientific experiments.

## Results

In this section, we present our main results for the understandability, reproducibility, and reuse of scientific experiments using a provenance-based semantic approach. We first precisely define “Reproducibility” and the related terms used throughout this paper. We then present the REPRODUCE-ME Data Model and ontology for the representation of scientific experiments along with their provenance information.

### Definitions

Reproducibility helps scientists in building trust and confidence in results. Even though different reproducibility measures are taken in different fields of science, it does not have a common global standard definition. Repeatability and Reproducibility are often used interchangeably even though they are distinct terms. Based on our review of state-of-the-art definitions of reproducibility, we precisely define the following terms [48] which we will use throughout this paper in the context of our research work inspired by the definitions [7, 49].

#### **Definition 1**

**Scientific Experiment**: A scientific experiment **E** is a set of computational steps **CS** and non-computational steps **NCS** performed in an order **O** at a time **T** by agents **A** using data **D**, standardized procedures **SP**, and settings **S** in an execution environment **EE** generating results **R** to achieve goals **G** by validating or refuting the hypothesis **H**.

#### **Definition 2**

**Computational Step**: A computational step **CS** is a step performed using computational agents or resources like computer, software, script, etc.

#### **Definition 3**

**Non-computational Step**: A non-computational step **NCS** is a step performed without using any computational agents or resources.

#### **Definition 4**

**Reproducibility**: A scientific experiment **E** composed of computational steps **CS** and non-computational steps **NCS** performed in an order **O** at a point in time **T** by agents **A** in an execution environment **EE** with data **D** and settings **S** is said to be reproducible, if the experiment can be performed to obtain the same or similar (close-by) results to validate or refute the hypothesis **H** by making variations in the original experiment **E**. The variations can be done in one or more of the following variables:

- Computational steps **CS**
- Non-Computational steps **NCS**
- Data **D**
- Settings **S**
- Execution environment **EE**
- Agents **A**
- Order of execution **O**
- Time **T**

#### **Definition 5**

**Repeatability**: A scientific experiment **E** composed of computational steps **CS** and non-computational steps **NCS** performed in an order **O** at a point in time **T** by agents **A** in an execution environment **EE** with data **D** and settings **S** is said to be repeatable, if the experiment can be performed with the same conditions of the original experiment **E** to obtain the exact results to validate or refute the hypothesis **H**. The conditions which must remain unchanged are:

- Computational steps **CS**
- Non-Computational steps **NCS**
- Data **D**
- Settings **S**
- Execution environment **EE**
- Agents **A**
- Order of execution **O**

#### **Definition 6**

**Reuse**: A scientific experiment **E** is said to be reused if the experiment along with the data **D** and results **R** are used by a possibly different experimenter **A** ^′^ in a possibly different execution environment **EE** ^′^ but with a same or different goal **G** ^′^.

#### **Definition 7**

**Understandability**: A scientific experiment **E** is said to be understandable when the provenance information (What, When, Where, Who, Which, Why, How) and the metadata used or generated in **E** are presented to understand the data **D** and results **R** of **E** by a possibly different agent **A** ^′^.

Understandability of scientific experiments is objective as the metadata is defined by the domain-specific community.

### **The REPRODUCE-ME data model and ontology**

The REPRODUCE-ME Data Model [50, 51] is a conceptual data model developed to represent scientific experiments with their provenance information. Through this generic data model, we describe the general elements of scientific experiments for their understandability and reproducibility. We collected provenance information from interviews and discussions with researchers from different disciplines and formulated them in the form of competency questions as described in the Methods section. The REPRODUCE-ME Data Model is extended from PROV-O [23] and P-Plan [33] and inspired by provenance models [15, 16].

**Fig. 2:**
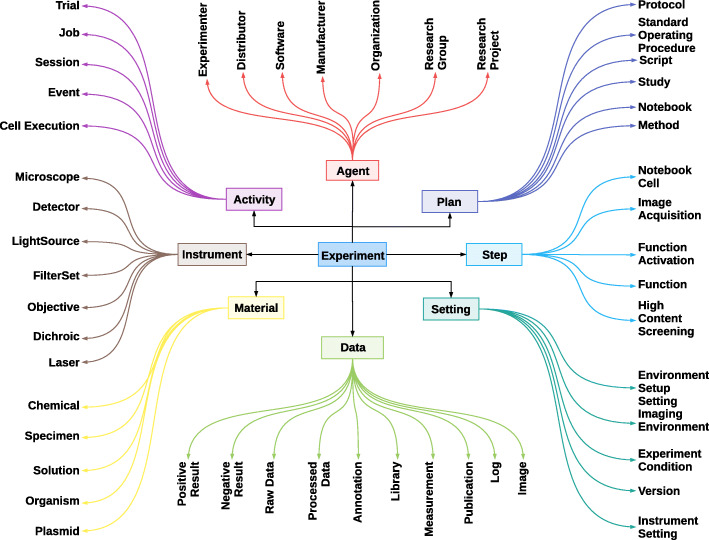
The expanded view of the REPRODUCE-ME data model used to represent a scientific experiment

Figure 2 presents the overall view of the REPRODUCE-ME data model to represent a scientific experiment.

The central concept of the REPRODUCE-ME Data Model is an *Experiment*. The data model consists of eight main components. They are Data, Agent, Activity, Plan, Step, Setting, Instrument, and Material.

#### **Definition 8**

Experiment is defined as a n-tuple *E* = (*Data, Agent, Activity, Plan, Step, Setting, Instrument, Material*)

where *E* is the Experiment; *Data* denotes the set of data used or generated in *E*; *Agent*, the set of all people or organizations involved in *E*; *Activity*, the set of all activities occurred in *E*; *Plan*, the set of all plans involved in *E*; *Step*, the set of steps performed in *E*; *Setting*, the set of all settings; *Instrument*, the set of all devices used in *E* and *Material*, the set of all physical and digital materials used in *E*.

We define each of the components of the model in detail. The definitions of the classifications of each component of the model are available in the documentation of the REPRODUCE-ME ontology [52] (see Additional file 1).

#### **Definition 9**

*Data* represents a set of items used or generated in a scientific experiment *E*.

Data is a key part of a scientific experiment. Though it is a broad concept, we need to narrow it down to specific details to model a scientific experiment for reproducibility or repeatability. Hence, the REPRODUCE-ME data model further categorizes the data. However, different instances of the same data type can belong to different categories. For example, an instance of a *Publication* from which a method or an algorithm is followed is annotated as *Input Data* and another instance of *Publication* could be annotated as *Result* of a study. We model *Data* as a subtype of *Entity* defined in the PROV data model. We classify the data as follows:

- Metadata
- Annotation
- Input Data
- Parameter
- Result
  - Final Result
  - Intermediate Result
  - Positive Result
  - Negative Result
- Raw Data
- Processed Data
- Measurement
- Publication
- Modified Version
- License Document
- Rights and Permissions Document

We use PROV-O classes and properties like *wasDerivedFrom*, *specializationOf*, *wasRevisionOf*, *PrimarySource* to describe the provenance of data, especially the transformation and derivation of entities.

#### **Definition 10**

*Agent* represents a group of people/organizations associated with one or many roles in a scientific experiment *E*.

Every agent is responsible for one or multiple roles associated with activities and entities of an experiment. For the reproducibility of scientific experiments, it is important to know the agents and their roles. However, it would be less significant for some experiments or disciplines to know all the agents involved. For example, the name of the manufacturer or distributor of a chemical substance/device is important in a life science experiment while it is less relevant for a computer scientist. Here, we present a list of relevant agents [54] based on our requirements directly or indirectly associated with a scientific experiment:

- Experimenter
- Manufacturer
- Copyright Holder
- Distributor
- Author
- Principal Investigator
- Contact Person
- Owner
- Organization
  - Research Project
  - Research Group
  - Funding Agency

#### **Definition 11**

*Activity* represents a set of actions where each action has a starting and ending time which involves the usage or generation of entities in a scientific experiment *E*.

Activity is mapped to *Activity* in the PROV-DM model. It represents a set of actions taken to achieve a task. Each execution or the trials of an experiment is considered as an activity that is also considered necessary to understand the derivation of the final output. Here we present the important attributes of activities of an experiment.

- Execution Order: The order of execution plays a key role in some systems and applications. For example, in a Jupyter Notebook, the order of the execution will affect the final and intermediate results because the cells can be executed in any order.
- Difference of executions: It represents the variation in inputs and the corresponding change in outputs in two different experiment runs. For example, two executions of a cell in a Jupyter Notebook can provide two different results.
- Prospective Provenance: It represents the provenance information of an activity that specifies its plan. e.g., a script.
- Retrospective Provenance: It represents the provenance information of what happened when an activity is performed. e.g., version of a library used in a script execution.
- Causal Effects: The causal effects of an activity denotes the effects on an outcome because of another activity. e.g., non-linear execution of cells in a notebook affects its output.
- Preconditions: The conditions that must be fulfilled before performing an activity. e.g., software installation prerequisites.
- Cell Execution: The execution/run of a cell of a computational notebook is an example of an activity.
- Trial: The various tries of an activity. For example, several executions of a script.

#### **Definition 12**

*Plan* represents a collection of steps and actions to achieve a goal.

The Plan is mapped to *Plan* in the PROV-DM and P-Plan model. Here, we classify the Plan as follows: (a) Experiment, (b) Protocol, (c) Standard Operating Procedure, (d) Method, (e) Algorithm, (f) Study, (g) Script, (h) Notebook.

#### **Definition 13**

*Step* represents a collection of actions that represents the plan for an activity.

A Step represents a planned execution activity and is mapped to *Step* in the P-Plan model. Here, we categorize Step as follows: (a) Computational Step, (b) Non-computational Step, (c) Intermediate Step, (d) Final Step.

#### **Definition 14**

*Setting* represents a set of configurations and parameters involved in an experiment.

Here, we categorize the Settings as follows: (a) Execution Environment, (b) Context, (c) Instrument Settings, (d) Computational Tools, (e) Packages (f) Libraries, (g) Software.

#### **Definition 15**

*Instrument* represents a set of devices used in an experiment.

In our approach, we focused onto the high-end light imaging microscopy experiments. Therefore, we add the terms which are related to microscopy to include domain semantics. The Instrument can be extended based on the requirements of an experiment. Here, we categorize the Instruments as follows: (a) Microscope, (b) Detector, (c) LightSource, (d) FilterSet, (e) Objective, (f) Dichroic, (g) Laser. This component could easily be extended to instruments from other domains.

#### **Definition 16**

*Material* represents a set of physical or digital entities used in an experiment.

We model the *Material* as a subtype of *Entity* defined in the PROV data model. Here, we provide some of the materials related to life sciences which are added in the data model: (a) Chemical, (b) Solution, (c) Specimen, (d) Plasmid. This could easily be extended to materials from other domains.

#### The REPRODUCE-ME ontology

To describe the scientific experiments in Linked Data, we developed a ontology based on the REPRODUCE-ME Data Model. The REPRODUCE-ME ontology, which is extended from PROV-O and P-Plan, is used to model the scientific experiments in general irrespective of their domain. However, it was initially designed to represent scientific experiments taking into account the life sciences and in particular high-end light microscopy experiments [50]. The REPRODUCE-ME ontology is available online along with the documentation [52]. The ontology is also available in Ontology Lookup Service [55] and BioPortal [53].

**Fig. 3:**
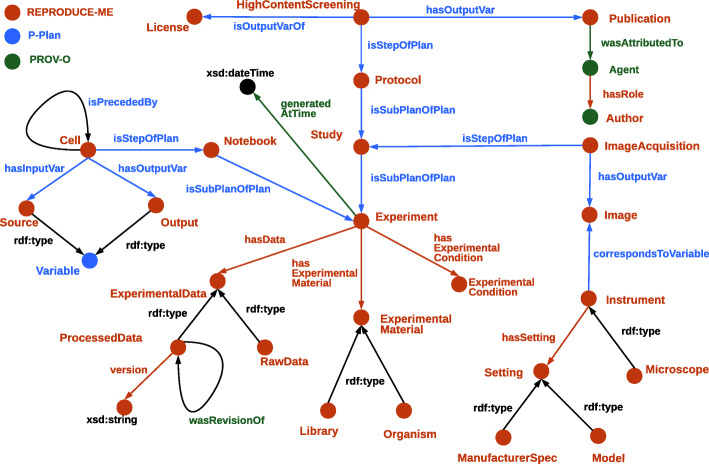
A scientific experiment depicted using the REPRODUCE-ME ontology [56]

Figure 3 shows an excerpt of the REPRODUCE-ME ontology depicting the lifecycle of a scientific experiment. The class *Experiment* which represents the scientific experiment conducted to test a hypothesis is modeled as a *Plan*. Each experiment consists of various steps and sub plans. Each step and plan can either be computational or non-computational. We use the object property *p-plan:isStepOfPlan* to model the relation of a step to its experiment and *p-plan:isSubPlanOfPlan* to model the relation of a sub plan to its experiment. The input and output of a step are modelled as *p-plan:Variable* which are related to the step using the properties *p-plan:isInputVarOf* and *p-plan:isOutputVarOf* respectively. The class *p-plan:Variable* is used to model each data element. For example, *Image* is an output variable of the *Image Acquisition* step which is an integral step in a life science experiment involving microscopy. The *Publication* is modeled as *ExperimentData* which in turn is a *p-plan:Variable* and *prov:Entity*. Hence, it could be used as an input or output variable depending on whether it was used or generated in an experiment. We use the properties *doi*^1^, *pubmedid*^2^, and *pmcid*^3^ to identify the publications.The concepts *Method*, *Standard Operating Procedure* and *Protocol*, which are modeled as *Plan* are added to describe the methods, standard operating procedures and protocols respectively. These concepts are linked to the experiment using the property *p-plan:isSubPlanofPlan*. The relationship between a step of an experiment and the method is presented using the object property *usedMethod*. The concepts *ExperimentalMaterial* and *File* are added as subclasses of a *prov:Entity* and *p-plan:Variable*. A variable is related to an experiment using the object property *p-plan:correspondsToVariable*. We could model the steps and plans and their input and output variables in this manner.

The role of Instruments and their settings are significant in the reproducibility of scientific experiments. The *Instrument* is modeled as a *prov:Entity* to represent the set of all instruments or devices used in an experiment. The configurations made in an instrument during the experiment is modeled as *Settings*. The parts of each *Instrument* are related to an *Instrument* using the object property *hasPart* and inverse property *isPartOf*. Each instrument and its parts have settings that are described using the object property *hasSetting*.

The agents responsible for an experiment are modeled by reusing the concepts of PROV-O. Based on our requirements to model agents in life-science experiments, we add additional specialized agents as defined in the REPRODUCE-ME Data Model to represent the agents directly or indirectly responsible for an experiment. We use the data property *ORCID* [57] to identify the agents of an experiment. We reuse the object and data properties of PROV-O to represent the temporal and spatial properties of a scientific experiment. The object property *prov:wasAttributedTo* is used to relate the experiment with the responsible agents. The properties *prov:generatedAtTime* and *modifiedAtTime* are used to describe the creation and modification time respectively.

To describe the complete path of a scientific experiment, it is important that the computational provenance is semantically linked with the non-computational provenance. Hence, in the REPRODUCE-ME ontology, we add the semantic description of the provenance of the execution of scripts and computational notebooks [58]. These are then linked with the non-computational provenance. We add the provenance information to address the competency question “What is the complete derivation of an output of a script or a computational notebook?”. Therefore, we present the components that we consider important in the reproducibility of scripts and notebooks to answer this question. Table 1 shows the components, their description and the corresponding terms that are added in the REPRODUCE-ME ontology to represent the complete derivation of scripts and notebooks. These terms are classified into prospective and retrospective provenance. The specification and the steps required to generate the results is denoted by prospective provenance. What actually happened during the execution of a script is denoted by retrospective provenance. We use each term to semantically describe the steps and sequence of steps in the execution of a script and notebook in a structured form using linked data without having to worry about any underlying technologies or programming languages.

**Table 1:** Overview of the ontology terms to model script and computational notebooks provenance

Component	Ontology term	Description	Provenance	Remarks
Script	*repr:Script*	Program or code that is used in a scientific experiment	Prospective	Subclass of *p-plan:Plan*
Function	*repr:Function*	A programming language code snippet	Prospective	Subclass of *p-plan:Plan*
Module	*repr:Module*	A part of a computer program or software which provides declarations and functions	Prospective	Subclass of *p-plan:Plan*
Module Version	*repr:Version*	The version of a module	Retrospective	Subclass of *repr:Setting*
Argument	*repr:Argument*	The parameter taken as an input, or declared/used in a script	Retrospective	Subclass of *p-plan:Variable*
Input	*repr:Input*	The variable used as an input to a script or a function	Retrospective	Subclass of *p-plan:Variable*
Output	*repr:Output*	The variable generated as an output of a script or a function	Retrospective	Subclass of *p-plan:Variable*
Programming Language	*repr:ProgrammingLanguage*	The programming language in which a script is written	Prospective	Subclass of *repr:Setting*
Programming Language Version	*repr:Version*	The version of the programming language in which a script is written	Retrospective	Subclass of *repr:Setting*
Operating System	*repr:OperatingSystem*	The operating system where the script is run	Retrospective	Subclass of *repr:Setting*
Operating System Version	*repr:Version*	The version of the operating system where the script is run	Retrospective	Subclass of *repr:Setting*
Author	*repr:Author*	The person who is the author of the script	Prospective	Subclass of *prov:Person*
Function Activation	*repr:FunctionActivation*	Denotes when a function is activated or run	Retrospective	Subclass of *p-plan:Step*
Trial	*repr:Trial*	Denotes a run or execution of a script	Retrospective	Subclass of *prov:Activity*
Start Time	*prov:startedAtTime*	Denotes the time when the script is started to execute	Retrospective	Data property
Finish Time	*prov:endedAtTime*	Denotes the time when the script finishes its execution	Retrospective	Data property
Experimenter	*repr:Experimenter*	Denotes the person who is executing the script	Retrospective	Subclass of *prov:Person*
Location	*prov:Location*	Denotes the location where the script is executed	Retrospective	Using *prov:atLocation*
Accessed File	*repr:File*	Denotes the files that are accessed during the script execution	Retrospective	Subclass of *p-plan:Variable*
Order of execution	*p-plan:isPrecededBy*	Denotes how the functions are executed inside a script	Retrospective	Object property
Experiment	*repr:Experiment*	Denotes the scientific experiment in which the script was used to perform data computation to produce result	Prospective	Subclass of *p-plan:Plan*
Notebook	*repr:Notebook*	A computational notebook used in an experiment	Prospective	Subclass of *p-plan:Plan*
Cell	*repr:Cell*	A multiline text input field in a computational notebook	Prospective	Subclass of *p-plan:Step*
Source	*repr:Source*	The input of each cell	Retrospective	Subclass of *p-plan:Variable*
CellExecution	*repr:CellExecution*	Denotes an execution of a cell	Retrospective	Subclass of *p-plan:Activity*

As shown in Table 1, the function definitions and activations, the script trials, the execution time of the trial (start and end time), the modules used and their version, the programming language of the script and its version, the operating system where the script is executed and its version, the accessed files during the script execution, the input argument and return value of each function activation, the order of execution of each function and the final result are used to describe the complete derivation of an output of a script.

**Fig. 4:**
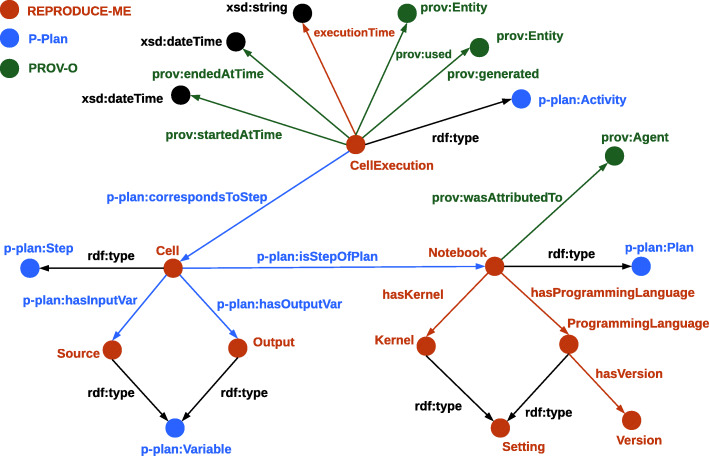
The semantic representation of a computational notebook [59]

The provenance of a computational notebook and its executions are depicted using the REPRODUCE-ME ontology in Fig. 4. The *Cell* is a step of *Notebook* and this relationship is described using *p-plan:isStepOfPlan*. The *Source* is related to *Cell* using the object property *p-plan:hasInputVar* and its value is represented using the property *rdf:value*. Each execution of a cell is described as *CellExecution* which is modeled as a *p-plan:Activity*. The input of each *Execution* is an *prov:Entity* and the relationship is described using the property *prov:used*. The output of each *Execution* is an *prov:Entity* and the relationship is described using the property *prov:generated*. The data properties *prov:startedAtTime*, *prov:endedAtTime*, and *repr:executionTime* are used to represent the starting time, ending time and the total time taken for the execution of the cell respectively.

To sum up, the REPRODUCE-ME ontology describes the non-computational and computational steps and plans used in an experiment, the people who are involved in an experiment and their roles, the input and output data, the instruments used and their settings, the execution environment, the spatial and temporal properties of an experiment to represent the complete path of a scientific experiment.

### Evaluation

**Fig. 5:**
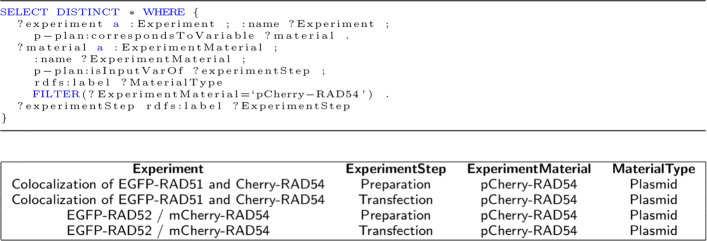
The steps involved in an experiment which used the Plasmid ‘pCherry-RAD54’

In this section, we apply the traditional ontology evaluation method by answering the competency questions through the execution of SPARQL queries. All questions mentioned in the Methods section could be answered by running the SPARQL queries over the provenance collected in CAESAR [56]. CAESAR (**C**oll**A**borative **E**nvironment for **S**cientific **A**nalysis with **R**eproducibility) is a platform for the end-to-end provenance management of scientific experiments. It is a software platform which is extended from OMERO [47]. With the integration of the rich features provided by OMERO and provenance-based extensions, CAESAR provides a platform to support the understandability and reproducibility of experiments. It helps scientists to describe, preserve and visualize their experimental data by providing the linking of the datasets with the experiments along with the execution environment and images [56]. It also integrates ProvBook [59], which captures and manages the provenance information of the execution of computational notebooks. We present here three competency questions with the corresponding SPARQL queries and part of the results obtained on running them against the knowledge base in CAESAR. The knowledge base consists of 44 experiments recorded in 23 projects by the scientists from the CRC ReceptorLight. The total size of the datasets including experimental metadata and images amount to 15GB. In addition to that, it consists of 35 imaging experiments from the IDR datasets [60]. The knowledge base consists of around 5.8 million triples. In our first question to get all the steps involved in an experiment which used a particular material, we showcase the answer using a concrete example, namely steps involving the Plasmid ‘pCherry-RAD54’. The corresponding SPARQL query and part of the results are shown in Fig. 5. As seen from Fig. 5, 2 experiments (Colocalization of EGFP-RAD51 and EGFP-RAD52 / mCherry-RAD54) use the Plasmid ‘pCherry-RAD54’ in the two different steps (‘Preparation’ and ‘Transfection’). The response time for this SPARQL query is 94ms.

**Fig. 6:**
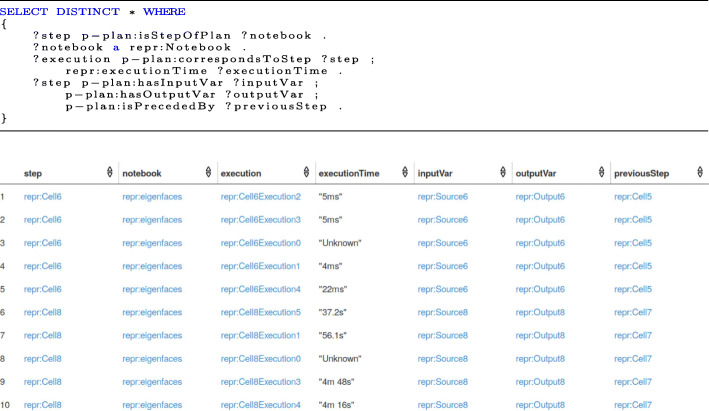
Complete path taken by a scientist for a computational notebook experiment: The corresponding SPARQL query and a part of results

The SPARQL query to answer the competency question ‘What is the complete path taken by a user for a computational notebook experiment’ and part of the results are shown in Fig. 6. The response time for this SPARQL query is 12ms.

**Fig. 7:**
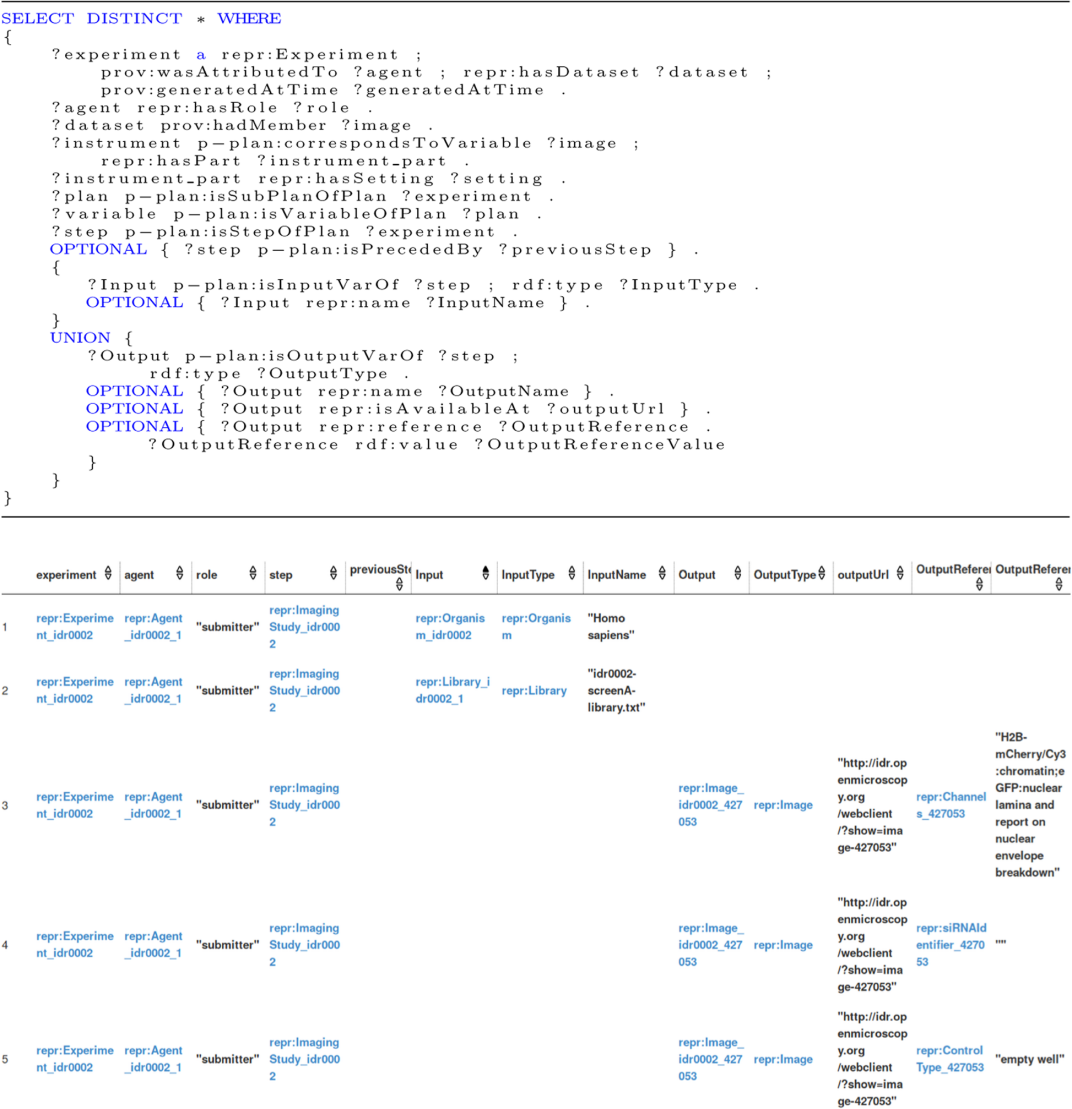
Complete path taken by a scientist for an experiment: The corresponding SPARQL query and a part of results

The SPARQL query to answer the competency question ‘What is the complete path taken by a user for a scientific experiment’ and its parts of results are shown in Fig. 7. This SPARQL queries a particular experiment called ’Focused mitotic chromosome condensation screen using HeLa cells’ with its associated agents and their role, the plans and steps involved, the input and output of each step, the order of steps, and the instruments and their setting. The results show that this query helps in getting all the important elements required to describe the complete path of an experiment. The experiment is linked to the computational and non-computational steps. It is possible that the query can be further expanded to query for all the elements mentioned in the REPRODUCE-ME Data Model. The response time for this SPARQL query is 824ms.

## Method

The design and development of REPRODUCE-ME Data Model and the ontology started with a use case driven approach. The first step in this work was to understand the current practices in science to perform and preserve scientific experimental data. We conducted several fruitful meetings with discussions among scientists throughout our work. To understand the experimental workflow of scientists from the university, lab visits were also conducted. The growing need for a framework for the description of experimental data for reproducibility and reuse in research groups in project consortiums was brought up to our attention through these meetings, interviews and lab visits. The results from these interviews and the recent study on the reproducibility crisis pointed out the necessity to address this problem starting from the bottom level.

We conducted a literature survey to understand the current state of the art on the approaches and tools that help the scientists towards reproducible research. The study pointed out that most of the works are based on the Scientific Workflow Management Systems [28] and the conservation of the scientific workflows [27]. Based on our understanding of scientific practices in the first step of our study, we identified that there are many experimental workflows that do not depend or require such complex scientific workflow management systems. There are many experimental workflows that are based on wet-lab activities and the additional analyses are done using scripts and other software. To address these workflows, we focused on linking all the non-computational data and steps with the computational data and steps to derive a path to the final results. The current state of the art approaches lack the interlinking of the results, the steps that generated them and the execution environment of the experiment in such scientific workflows.

To describe the complete path of a scientific experiment, we reviewed the use of semantic web technologies. We first studied the existing provenance models and how they can be used to design a conceptual model in describing experiments. We aimed to reuse the existing standard models and extend them for this research. Therefore, we selected the provenance data model, PROV-O [23] which closely meets our requirements, and provides the support to interoperably extend it further for specific domain needs. We developed our conceptual model by extending PROV-O to describe scientific experiments. We used another provenance model [33] to describe the steps and their order in detail. Reusing PROV-O and P-Plan, we designed the REPRODUCE-ME data model and the ontology to represent the complete path of scientific experiments [50, 51].

We used a collaborative approach to design and develop the ontology [61–63]. In the *Preparation* phase, we identified the requirements of the REPRODUCE-ME ontology by defining the design criteria, determining the boundary conditions and deciding upon the evaluation standards. We first narrowed down the domain of the ontology to the scientific experiments in the microscopy field. We defined the aim of developing the ontology to semantically represent the complete path of a scientific experiment including the computational and non-computational steps along with its execution environment. We determined the scope of the ontology to use it in the scientific data management platforms as well as the scripting tools that are used to perform computational experiments. The end-users of the ontology were identified to be the domain scientists from life sciences who want to preserve and describe their experimental data in a structured format. The ontology could provide a meaningful link between the data, intermediate and final results, methods and execution environment which will help the scientists to follow the path used in experiments. We defined the competency questions based on the requirements and interviews with the scientists. We created an Ontology Requirement Specification Document (ORSD) which specifies the requirements that the ontology should fulfill [64] and also the competency questions (see Additional file 2). The OWL 2 language was used for knowledge representation and serves as a baseline for all the axioms included in the ontology. The DL expressivity of REPRODUCE-ME ontology is *SRIN(D)*, allowing role transitivity, complex role inclusion axioms, inverse relations, cardinality restrictions, and the use of datatype properties. These relations are helpful to infer new information from the ontology using reasoning in description logic. The ontology is aimed at RDF applications that require scalable reasoning without sacrificing too much expressive power.

In the *Implementation* phase, we used *Protege* [65] as the ontology tool editor for the development of the ontology. We used RDF/XML for the serialization of the ontology. We used the same CamelCase convention which is also used in PROV-O and P-Plan. The prefix used to denote the ontology is “repr”. The namespace of the ontology is “https://w3id.org/reproduceme#”.

In the *Annotation* phase, we added several annotations to the ontology to capture the provenance of the ontology. It includes the creator, creation and modified time, etc. In the *Documentation and Publication*, we used the WIDOCO tool [66] to document the ontology. The ontology uses persistent URLs to make the ontology terms dereferenceable. The ontology is available in RDF/XML, TTL or N3 serializations. In the *Validation* phase, we used the OOPS tool [67] to validate the ontology. The common pitfalls detected during its development were corrected as and when they were found.

We used application-based and user-based evaluation to evaluate our approach. Scientists are involved in the evaluation and also being the users of our solution. In the application-based evaluation, ontologies are used in systems to produce good results on a given task [68]. The evaluation was done on CAESAR [56] which is hosted on a server (installed with CentOS Linux 7 and with x86-64 architecture) at the University Hospital Jena. The REPRODUCE-ME ontology was evaluated in the context of scientific experiments related to high-end light microscopy. Scientists from B1 and A4 projects of ReceptorLight documented experiments using confocal patch-clamp fluorometry (cPCF), Förster Resonance Energy Transfer (FRET), PhotoActivated Localization Microscopy (PALM) and direct Stochastic Optical Reconstruction Microscopy (dSTORM) as part of their daily work. In 23 projects, a total of 44 experiments were recorded and uploaded with 373 microscopy images generated from different instruments with various settings using either the desktop client or webclient of CAESAR (Accessed 21 April 2019). We also used the Image Data Repository (IDR) datasets [60] with around 35 imaging experiments [69] for our evaluation to ensure that the REPRODUCE-ME ontology can be used to describe other types of experiments as well. The scientific experiments along with the steps, experiment materials, settings, and standard operating procedures were described using the REPRODUCE-ME ontology using the Ontology-based Data Access Approach (OBDA) [70]. A knowledge base of different types of experiments was created from these two sources.

We used the REPRODUCE-ME ontology to answer the competency questions using the scientific experiments documented in CAESAR for its evaluation. The competency questions which were translated into SPARQL queries by computer scientists were executed on our knowledge base which consists of linked data in CAESAR. The correctness of the answers to these competency questions was evaluated by the domain experts.

## Discussion

Each of the competency questions addressed the different elements of the REPRODUCE-ME Data Model. The ontology was also evaluated with different variations in the competency questions. Answering the competency questions using SPARQL queries show that some experiments documented in CAESAR had missing provenance data on some of the elements of REPRODUCE-ME Data Model like time, settings, etc. In addition to that, the output of the query for finding the complete path of scientific experiment results in many rows in the table. Therefore, the response time could exceed the normal query response time and result in server error from the SPARQL endpoint in some cases where the experiment has various inputs and outputs with several executions. To overcome this issue, the queries were split and their results were combined in CAESAR. The entities, agents, activities, steps, and plans in CAESAR are grouped to help users visualize the complete path of an experiment.

Currently, scientists from the life sciences are not familiar with writing their own SPARQL queries. However, scientists must be able to see the answers from these competency questions and explore the complete path of a scientific experiment. The visualization module in CAESAR which uses SPARQL and linked data in the background, provides the visualization of the provenance graph of each scientific experiment. The visualization of the experimental data and results using CAESAR supported by the REPRODUCE-ME ontology helps the scientists without worrying about the underlying technologies. The competency questions, the RDF data used for the evaluation, the SPARQL queries, and their results are publicly available [71].

## Conclusion

In this article, we presented the REPRODUCE-ME Data Model and the ontology to describe the provenance of scientific experiments. We developed the REPRODUCE-ME by extending existing standards like PROV-O and P-Plan. We provided a precise definition of reproducibility and related terms which present a basis for the ontology. We studied computational reproducibility and added concepts and relations to describe provenance of computational experiments using scripts and Jupyter Notebooks.

We evaluated our approach by integrating the ontology in CAESAR and answering competency questions over the knowledge base of scientific experiments. The provenance of the scientific experiments are captured and semantically represented using the REPRODUCE-ME ontology. The computational data and steps are linked to the non-computational data and steps to represent the complete path of the experimental workflow.

Aligning the REPRODUCE-ME ontology with other possible existing ontologies to describe provenance of scientific experiments is one of the future area of research. We plan to use vocabularies like DCAT [43] to describe provenance of the datasets.

## Supplementary Information

**Additional file 1** The REPRODUCE-ME ontology. A supplemental document containing the REPRODUCE-ME ontology in OWL format (reproduce-me.owl) is available for this manuscript.

**Additional file 2** The REPRODUCE-ME ORSD. A supplemental document containing the REPRODUCE-ME Ontology Requirement Specification Document (REPRODUCE-MEORSD.pdf) is available for this manuscript.

## Data Availability

The information on the results, code and data is available at https://w3id.org/reproduceme/research. The REPRODUCE-ME ontology is available at https://w3id.org/reproduceme/. The source code of CAESAR is available at https://github.com/CaesarReceptorLight. The source code of ProvBook is available at https://github.com/Sheeba-Samuel/ProvBook.

## Abbreviations

CAESAR: CollAborative environment for scientific analysis with reproducibility
SWfMS: Scientific workflow management system
OWL: Web ontology language
RDF: Resource description framework
SPARQL: SPARQL protocol and RDF query language
FAIR: Findable, accessible, interoperable, reusable
CRC: Collaborative research center
W3C: World wide web consortium
OPMW: Open provenance model for workflows
OBI: Ontology for biomedical investigations
OMERO: Open microscopy environment remote objects
OME: Open microscopy environment
ORCID: Open researcher and contributor ID
ROI: Region of interest
OBDA: Ontology-based data access
CPCF: Confocal patch-clamp fluorometry
FRET: Förster resonance energy transfer
PALM: PhotoActivated localization microscopy
DSTORM: Direct stochastic optical reconstruction microscopy
IDR: Image data repository

## Footnotes

1 https://www.doi.org/

2 https://www.ncbi.nlm.nih.gov/pubmed/

3 https://www.ncbi.nlm.nih.gov/pmc/
