# Supplementary material for: End-to-End provenance representation for the understandability and reproducibility of scientific experiments using a semantic approach
Source: J Biomed Semantics. 2022 Jan 6;13:1. doi: 10.1186/s13326-021-00253-1 (PMC8734275; doi:10.1186/s13326-021-00253-1)
Supplement: Supplementary file 2 — Additional file 2 The REPRODUCE-ME ORSD. A supplemental document containing the REPRODUCE-ME Ontology Requirement Specification Document (REPRODUCE-MEORSD.pdf) is available for this manuscript. [file 13326_2021_253_MOESM2_ESM.pdf]

The REPRODUCE-ME Ontology Requirements Specification Document

The REPRODUCE-ME Ontology Requirements Specification Document
**1. Purpose**
The purpose of this ontology is to represent the provenance of a scientific experiment to enable end-to-end reproducibility.
**2. Scope**
The ontology has to focus on the computational and non-computational processes of an experiment and the data used and generated in an experiment.
**3. Implementation Language**
The ontology will be implemented in OWL language.
**4. Intended End-Users**
User 1. Scientist aiming to track the provenance of scientific experiments.
User 2. Scientist aiming for end-to-end reproducibility of scientific experiments.
User 3. Scientist aiming to track the provenance of execution of scripts.
User 4. Scientist aiming to track the provenance of execution of Computational notebooks.
User 5. Scientist aiming to describe light microscopy imaging experiments.
**5. Intended Uses**
Use 1. Describe the provenance of scientific experiments.
Use 2. Describe the computational experiments conducted using scripts.
Use 3. Describe the computational experiments conducted using interactive notebooks.
Use 4. Describe the steps and the execution environment of experiments.

Table 1: The REPRODUCE-ME ORSD Slots 1-5

**6. Ontology Requirements**
**a. Non-functional Requirements**
NFR 1. The ontology must be published on the Web with an open and non-commercial license.
NFR 2. The ontology must be written in English.
NFR 3. The ontology must follow the Camel Case convention.
NFR 4. The ontology must be available via its namespace URI with human-readable documentation and machine-readable structured data using content negotiation.<br>NFR 5. The ontology must reuse other ontologies if required.
**b. Functional Requirements: Groups of Competency Questions**
CQ1. What are the input and output variables of an experiment?<br>CQ2. Which are the methods and standard operating procedures used?<br>CQ3. Which are the files and materials that were used in a particular step?<br>CQ4. Which are the steps involved in an experiment which used a particular material?<br>CQ5. Which are the instruments that are associated with an experiment and their settings when the output was generated?<br>CQ6. Which are the agents directly or indirectly responsible for an experiment?<br>CQ7. Who created this experiment and when? Who modified it and when?<br>CQ8. Which are the publications or external resources that were referenced?<br>CQ9. What is the complete path taken by a scientist for an experiment?<br>CQ11. What is the complete path taken by a user for a computational notebook experiment?<br>CQ12. What is the sequence of steps in the execution of a computational notebook?<br>CQ13. How many trials were performed for a particular cell in a computational notebook?<br>CQ14. How long it took for a particular trial of a computational notebook?<br>CQ15. What was the source for a particular trial of a computational notebook?<br>CQ16. What was the output for a particular trial of a computational notebook?<br>CQ17. Who are the agents responsible for the execution of a computational notebook?<br>CQ18. When was a particular trial of a computational notebook last executed?<br>CQ19. What are the environmental attributes of a notebook execution?<br>CQ20. What is the sequence of steps in the execution of a script?<br>CQ21. Which are the steps that invoke a particular module?<br>CQ22. Which are the environmental attributes in the execution of a script?<br>CQ22. List the user, the operating system, the processor, programming language version, the working directory associated with the execution of a script.<br>CQ24. What is the complete derivation of a script output?

Table 2: The REPRODUCE-ME ORSD Ontology Requirements

7. Pre-Glossary of Terms					
**a. Terms from Competency Questions + Frequency**					
Experiment	7	Step	6	Computation	8
Output	5	Input	2	Particular	8
Script	4	Notebook	8	Result	1
Setting	1	Complete	3	Execution	6
Trial	5	Sequence	2	Material	2
Agent	2	Environment	2	Instrument	1
File	1	Resource	1	Path	2
Attribute	2	Use	3	Responsible	2
Publication	1	Parameter	1	Procedure	1
Method	1	Version	1	Generate	2
**b. Terms from Answers + Frequency**					
Experiment	15	File	10	Code	4
Image	8	Plasmid	2	Protein	3
Microscope	4	Vector	3	Person	7
Setting	8	Solution	4	Data	6
Execution	6	Software	4	Material	6
Metadata	7	Format	3	Measurement	5
Time	2	Group	3	Instrument	3
Environment	2	Sample	2	Hardware	2
Result	7	Temperature	2	Project	1
Publication	4	Document	4	Cell	1
Version	3	Processed	2	Raw	1
**c. Objects**					
No objects were identified.					

Table 3: The REPRODUCE-ME ORSD Pre-Glossary of Terms
